# Retro-Active Emotion: Do Negative Emotional Stimuli Disrupt Consolidation in Working Memory?

**DOI:** 10.1371/journal.pone.0169927

**Published:** 2017-01-19

**Authors:** Güven Kandemir, Elkan G. Akyürek, Mark R. Nieuwenstein

**Affiliations:** Experimental Psychology, University of Groningen, Groningen, The Netherlands; University of Verona, ITALY

## Abstract

While many studies have shown that a task-irrelevant emotionally arousing stimulus can interfere with the processing of a shortly following target, it remains unclear whether an emotional stimulus can also retro-actively interrupt the ongoing processing of an earlier target. In two experiments, we examined whether the presentation of a negative emotionally arousing picture can disrupt working memory consolidation of a preceding visual target. In both experiments, the effects of negative emotional pictures were compared with the effects of neutral pictures. In Experiment 1, the pictures were entirely task-irrelevant whereas in Experiment 2 the pictures were associated with a 2-alternative forced choice task that required participants to respond to the color of a frame surrounding the pictures. The results showed that the appearance of the pictures did not interfere with target consolidation when the pictures were task-irrelevant, whereas such interference was observed when the pictures were associated with a 2-AFC task. Most importantly, however, the results showed no effects of whether the picture had neutral or emotional content. Implications for further research are discussed.

## Introduction

Research on the effects of emotional stimuli has provided ample evidence showing that the appearance of an emotional stimulus can lead to momentary distraction from the task at hand. For instance, studies on eye-witness testimonies suggest that the appearance of a weapon in a crime results in less accurate testimonies [1, 2], whereas studies on visual search have shown that the presence of an emotional stimulus reduces search efficiency [3], and studies on memory have shown that the appearance of an emotional stimulus can lead to forgetting of previously memorized stimuli [4]. In explaining these effects, it is commonly assumed that the processing of emotional stimuli is prioritized over neutral stimuli [5, 6]. Subcortical pathways including projections from lateral geniculate nucleus, pulvinar, superior colliculus are thought to be playing a role in this [7]. Additionally, feedback routes from amygdala [8], orbitofrontal cortex [9] and insula [10] to visual cortex are thought to enhance the activation for emotional stimuli, giving such stimuli a higher probability for attention allocation [11]. This attentional priority for emotional stimuli entails that such stimuli capture attention [12] and such attentional capture can hinder task performance when task irrelevant emotional stimuli are present during the execution of a particular task [13].

Effects of emotional stimuli on task performance have also been shown in the temporal domain, particularly in studies on the attentional blink, in which observers are asked to identify two targets embedded in a sequence of successive distractors that are presented for ~100 ms each. If the stimulus onset asynchrony (SOA) between the two targets is below ~600 ms, the second target is often missed [14]. Since sensory encoding of a stimulus is thought to be completed within 100 ms [15–19], the relatively long-lasting attentional blink is generally thought to reflect the time course of the second stage of processing required to consolidate the first target’s representation in working memory [20, 21].

In examining the influence of emotional stimuli, studies using the attentional blink have yielded several important insights (e.g., [22–25]). For instance, it was shown that when the second target is an emotional stimulus, the attentional blink is attenuated [26, 27]. Furthermore, it has also been found that the attentional blink is increased in magnitude and duration when the first target is a taboo or sexual word [28], suggesting that the arousing nature of emotional stimuli can increase the interference caused by the processing of such stimuli. Aside from demonstrating that targets with emotional significance are processed differently than neutral targets, studies on the attentional blink have also provided evidence that emotional stimuli may capture attention even if they are task-irrelevant. For example, Most and colleagues found that an attentional blink could be induced by a task irrelevant emotional stimulus [25]. In this study, a sequence of landscape and architectural pictures was shown, of which the target picture was rotated to left or right. The task for the participant was to indicate the direction of rotation and in some trials a critical distracter could be presented 200 ms prior to the target. This critical distracter was either a neutral, a negative or a scrambled version of a negative picture. The results showed reduced accuracy in discriminating target rotation when the target was preceded by an emotional picture relative to when it was preceded by a scrambled or a neutral picture. Thus the capture of attention by emotionally relevant stimuli directly caused an attentional blink.

Findings similar to those of Most et al. [25] were reported by Arnell and colleagues, who showed a sexual, negative, threatening, positive, or neutral word prior to the appearance of a target [29]. The results of this study revealed that when the target appeared shortly after a distracter which was a sexual word, target identification was impaired. This was not the case when a negative, positive, threatening or neutral word was shown prior to the target. Further analyses revealed that the extent of arousal of the stimuli correlated negatively with the accuracy in target identification. Additionally, by using a memory checklist for distracters, Arnell and colleagues showed that the attentional capture by arousing stimuli resulted in further processing of these task irrelevant items, such that memory for arousing words was significantly higher than for other distracter types.

While there is thus considerable evidence to suggest that the appearance of an emotionally arousing stimulus can capture attention and thereby cause or exacerbate an attentional blink effect, it remains unclear if and how an emotional stimulus might influence the on-going consolidation of an earlier shown stimulus. Previous evidence exists that attentional capture by task-irrelevant emotional stimuli suppresses not only attentional ERP components of subsequently presented target stimuli, but also memory-related ones, such as the P3, which occurs relatively late and is long-lasting [30]. There is furthermore one prior finding that suggests that emotional stimuli can influence the processing of an earlier target. In a single-target identification task similar to that used by Most et al. [25], Most and Junge [31] showed a negative emotional picture just after the target picture. The results showed that memory for the target was impaired when the negative emotional picture followed the target by 100 ms, whereas it was found to be enhanced when the emotional picture was presented 200 ms after the target. The aim of the current study was to examine these retroactive memory effects in closer detail.

One paradigm that appears especially useful for studying how emotional stimuli might influence the processing of an earlier shown stimulus is the retroactive interference paradigm used by Nieuwenstein and Wyble [32]. In this paradigm, a to-be-remembered stimulus consisting of a string of four letters is shown briefly and followed by a 2-alternative forced choice task (2-AFC) in some of the trials. The results of this type of task show retroactive interference during the on-going memory consolidation of the first target, such that target recall is reduced when the 2-AFC task follows the target within less than 600 ms. Nieuwenstein and Wyble explained this retroactive interference effect in terms of the episodic simultaneous type serial token model (eSTST), which is a neural network model that can simulate object recognition, attentional selection, and working memory consolidation. According to this model, sensory representations that attract attention are encoded into working memory by being assigned to episodic working memory representations. This involves a time-consuming binding process which takes up to several hundred milliseconds to be completed, thus explaining the gradual reduction in retroactive interference with increasing SOA. To explain how consolidation may be disturbed, the eSTST model assumes that, to ensure the protection of on-going consolidation of a target in working memory, the allocation of attention to new information is suppressed. Accordingly, the occurrence of retroactive interference requires that a stimulus breaks through this state of attentional suppression to disturb the ongoing consolidation of a preceding target, and Nieuwenstein and Wyble reasoned that the requirement to respond rapidly to the 2-AFC task meets this requirement, thus leading to the disruption of the on-going consolidation process.

Considering that emotionally arousing stimuli are known to capture attention [13, 25, 29], it follows that an emotional stimulus would be more likely to break-through the assumed attentional suppression and interfere with on-going consolidation than a neutral stimulus. To address this matter, we examined how the appearance of a picture with neutral or negative emotional contents would affect recall accuracy of a preceding target in a paradigm similar to the retroactive interference paradigm of Nieuwenstein and Wyble [32]. We expected negative emotional pictures to have a bigger effect in comparison to neutral pictures due to the attentional salience of emotional stimuli [3, 33].

## Methods Experiment 1

### Participants

The study was conducted in accordance with the Declaration of Helsinki and approved beforehand by the Ethical Committee Psychology at the University of Groningen (approval number 14075-NE). Eighteen psychology students volunteered for this experiment in return for course credit, and gave written informed consent. The sample consisted of fifteen female and three male participants with a mean age of 20.44 years (SD = 1.50 years).

### Materials & Stimuli

**Fig 1:**
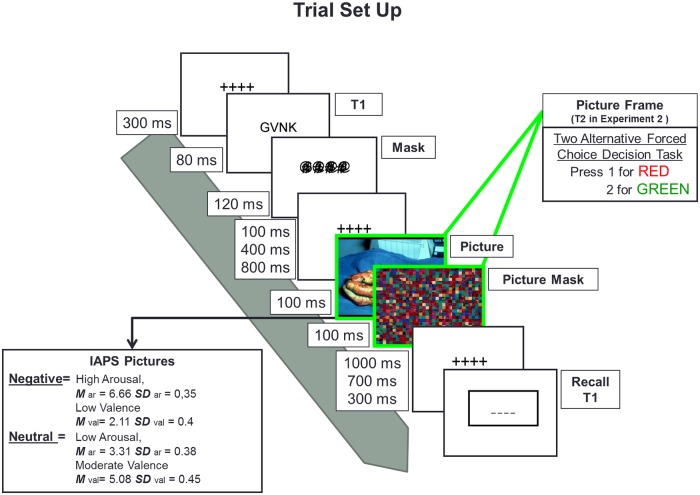
Experiment 1 & 2 picture-present trial procedure.

The experiment was programmed using E-Prime 2.0 [34], and it was run on a computer equipped with a 19-inch CRT monitor that had a resolution of 1024 by 768 pixels and a 100 Hz refresh rate. As shown in Fig 1, a trial comprised the presentation of a target (T1), which consisted of four letters followed by a mask, a blank interval of varying duration, and, in 50% of the trials, a picture that depicted either neutral or negative emotional content, and an ensuing mask. The four letters were selected at random from the English Alphabet, excluding “W”, “M” and all vowels. The letters were shown at the center of a white background in black Helvetica 20 pt. font. The mask for the letters consisted of three strings of four superimposed “#”, “$” and “@” symbols, thus yielding a pattern mask that included most features of the previously shown letters. The font for the mask was Courier New (again 20 pt. size). The picture that could follow the presentation of the letters and their mask was drawn from a set of 114 pictures, obtained from the International Affective Picture System (IAPS; [35]). Of these 114 pictures, 57 were emotionally arousing negative pictures and 57 were neutral pictures. Negative pictures were selected on the basis of their valence and arousal levels, as established on a Likert scale of 1 to 9, with the criteria for inclusion being that the pictures should have a rating between 6 and 9 for arousal and between 1 and 3 for valence, with valence ratings of 1 representing the highest negativity for the picture. For the 57 pictures we selected, the mean valence rating was 2.11 (SD = 0.45) and the mean arousal rating was 6.67 (SD = 0.33). Emotionally negative pictures contained gruesome human or animal images (e.g., mutilations, corpses, etc.), threats (e.g., guns, assault, etc.), or disasters (e.g., accidents, scenes of war, etc.). The neutral pictures had arousal ratings between 1 and 3 and valence ratings between 4 and 6, with a mean valence of 5.08 (SD = 0.45) and a mean arousal of 3.31 (SD = 0.38). The neutral pictures were selected so that they matched the contents of the negative pictures such that both pictures had approximately the same human or animal content in the same position. The pictures were presented at the center of the screen and they all had a size of 384 x 512 pixels. The mask presented after a picture was drawn randomly from a set of 14 pictures that consisted of a scrambled version of a picture with neutral content. Scrambled pictures were made with Irfanview Thumbnails Ink. Image editor by dividing the original pictures into 32x32 pixel squares and randomly mixing these squares using the scramble filter plug-in downloaded from www.telegraphics.com.au/sw/product/scramble. The size of the mask was the same as the picture. For practice trials, a different set of pictures from IAPS was used (neutral pictures N = 14, M valence = 4.93, M arousal = 2.83; emotional pictures N = 14, M valence = 1.86; M arousal = 6.11), to avoid familiarization.

### Design

The experiment consisted of a 3 x 3 within-subjects design, with stimulus onset asynchrony (SOA: 300, 600, 1000 ms) and the nature of the picture stimulus (picture condition; none, negative, neutral) as factors. Half of the trials did not include a picture, with the remaining 50% of the trials being equally likely to contain a negative or a neutral picture. During the experiment, each picture was used only once and the selection of pictures was done in such a way that if a participant encountered a negative picture with certain contents at a particular SOA, then he or she would encounter a neutral picture with matching contents at the same SOA. In addition, the pictures were selected in such a way that they were shown equally often across different SOA across the different participants, thus ensuring that any effect of SOA could not be due to the frequency with which pictures appeared at the different SOAs. In the experiment, there were 19 trials per SOA and picture condition, thus yielding a total of 228 trials. The experiment was preceded by 6 practice trials.

Throughout our analyses we used ANOVA to test our observations. In case of a violation of the sphericity assumption, Greenhouse-Geisser correction was applied and the adjusted degrees of freedom are reported. Aside from conducting null-hypothesis significance tests, we also conducted Bayesian analyses to further characterize the evidence in favor of both significant and non-significant effects. Using Bayes factors, one can quantify the extent to which the acquired data support the existence (H_1_) or absence (H_0_) of an effect, with a continuous measure that expresses the ratio of the likelihood of the data under these respective hypotheses [36–38]. A key advantage of this method is that it allows for an assessment of the evidence for both H_1_ and H_0_, whereas the traditional approach of significance testing only allows one to test for evidence against the null. Furthermore, it has been shown that, compared to significance tests, Bayes factors provide a more robust test of the acquired evidence as significance tests tend to overestimate the evidence against H_0_ [39]. In calculating Bayes factors, we used the default Cauchy prior settings in the JASP package [40] and in reporting the results of the Bayes factors analyses, we expressed the evidence in favor of H_1_ in terms of *BF*_*10*_ and we expressed the evidence in favor of H_0_ in terms of *BF*_*01*_. For all *BF*’s, values greater than 1 signify evidence in favor of one hypothesis over the other and in characterizing the obtained *BF*’s we followed the nomenclature provided in the JASP statistical package, which considers *BF*’s of 1–3 as anecdotal evidence; 3–10 as moderate evidence; 10–30 as strong evidence, and 30–100 as very strong evidence.

### Procedure

On arrival, participants were informed about the nature of the experiment and the stimuli via a couple of example trials. The instruction given to the participants was to memorize the letters shown, which might be followed by a picture and its mask in some trials. Participants were told to keep attention focused on the screen until the box appeared in which they could type in the letters.

Each trial began with the appearance of a row of four crosses at the center of the screen, to guide the participant’s gaze, and the presentation of the target stimuli began 500 ms after the participant pressed the spacebar. The target letters were displayed for 80 ms, and followed in turn by a mask, which appeared for 120 ms. In the conditions in which a picture was present, the mask for the letters was followed by a blank inter-stimulus interval of 100, 400 or 800 ms, and then the appearance of a picture for 100 ms and its mask for 100 ms. Following the picture mask, the row of crosses was again shown for 300, 700 or 1000 ms, depending on SOA, so as to keep the time between the onset of target letters and the moment of recall fixed at 1500 ms. In the control condition without a picture, the appearance of the mask for the letters was followed by a blank screen for a period that matched the duration of the period between mask and moment of recall in the conditions in which the picture was present. A trial was completed after the participant typed in the letters he or she remembered.

## Results

**Fig 2:**
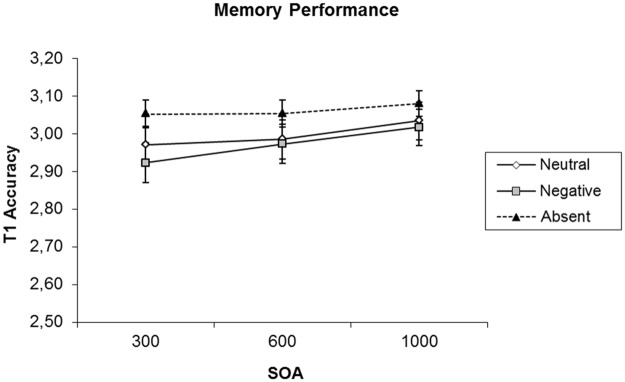
T1 accuracy by SOA in Experiment 1. Separate lines plot different picture conditions. The error bars show standard error of the mean.

The first analysis concerned T1 recall, to determine if the total number of letters recalled for T1 depended on SOA and picture condition. The results, shown in Fig 2, revealed a significant main effect of picture condition, *F*(2, 34) = 4.54, *MSE* = 0.03, *p* = 0.02, *η*^*2*^ = 0.21, *BF*_*10*_ = 6.17, but no main effect of SOA, nor of the interaction of SOA and picture condition, *F*(2, 34) = 1.87, *MSE* = 0.03, *p* = 0.17, *BF*_*01*_ = 2.18, and *F*(4, 68) = 0.37, *MSE* = 0.02, *p* = 0.83, *BF*_*01*_ = 15.63, respectively. To examine the main effect of picture condition content in closer detail, we conducted pairwise comparisons with Bonferroni correction. These comparisons revealed that recall performance was better in the picture-absent condition than in both picture-present conditions (absent vs. neutral, *t(53)* = 2.83, *p* = 0.03, *BF*_*10*_ = 4.24; absent vs. emotional, *t(53)* = 2.68, *p* = 0.05, *BF*_*10*_ = 22.00). Importantly, however, emotional content did not have a statistically significant effect on recall, as the difference between the emotional and neutral pictures was non-significant (neutral vs. emotional, *t(53)* = 0.74, *p* = 1, *BF*_*01*_ = 4.17).

## Discussion of Experiment 1

The results of Experiment 1 show that the appearance of a task-irrelevant picture with either neutral or negative emotional contents interfered with the ability to recall an earlier shown target. Importantly, however, this effect did not depend on whether the picture had neutral or negative emotional contents, and it did not interact with SOA, indicating that the interference did not depend on how much time was available for target consolidation prior to the appearance of the picture. Accordingly, it can be concluded that a task-irrelevant emotional stimulus does not interrupt the consolidation of an earlier shown target as the effects of such an interruption would be expected to be larger at shorter SOAs. In Experiment 2, we examined whether a difference between neutral and emotional pictures would be observed when these pictures are task-relevant, in the sense that they were associated with a 2-AFC task similar to that used by Nieuwenstein and Wyble [32]. We thus replicated Experiment 1 with the difference being that now, the pictures were made task-relevant by showing them in either a red or a green outline square, and asking participants to respond as quickly as possible to indicate whether the color of the outline square was red or green.

## Methods Experiment 2

### Participants

Experiment 2 followed the same procedures as Experiment 1. The participants for Experiment 2 were 24 new psychology students from the University of Groningen who volunteered for this experiment in return for course credits. There were nine male and fifteen female participants, with a mean age of 20.88 years (SD = 1.85 years).

### Materials & Stimuli

The materials and stimuli used in Experiment 2 were identical to those used in Experiment 1, with the exception that the picture and mask shown were now always surrounded by a red or a green outline frame that had a thickness of 10 pixels. Because the framed picture was now task-relevant, we will henceforth refer to it as the second target stimulus (T2).

### Design

The design of Experiment 2 was identical to the design of Experiment 1.

### Procedure

The procedure of Experiment 2 was identical to the procedure in Experiment 1, with the exception that in Experiment 2, participants needed to respond as quickly as possible to T2 by indicating whether the outline frame surrounding the picture and its mask was red or green. Following their response to T2, the participants were asked to type in the letters they remembered, which was the same task associated with T1 as before. In the condition without a picture, that is, without T2, the presentation of T2 and its mask were replaced by blank screens, and we inserted an additional blank retention interval of 800 ms prior to report. The inclusion of the blank retention interval served to ensure that the retention interval in the T2-absent condition would be comparable to that in the T2-present conditions in which participants reported the letters after they had responded to T2.

## Results

The analyses of T1 recall accuracy included trials in which T2 was absent, and those trials in which T2 was present and responded to accurately. In addition, we first performed an outlier exclusion procedure based on the response times for T2-present trials. This procedure entailed that we first excluded trials in which the response time was shorter than 200 ms or longer than 2000 ms, and we then submitted the remaining trials to the recursive outlier exclusion procedure described by Van Selst and Jolicoeur [41]. As a result of this procedure, 11% of the T2-present trials were omitted from the analysis of T1. Of these trials, 5.5% of the pictures was neutral, and 5.8% was negative. Exclusion of the trials did not depend on picture type, as determined by a paired t-test comparing the percentage of excluded trials in the conditions with negative and neutral pictures, 2-tailed *t*(23) = −0.84, *p* = 0.41.

**Fig 3:**
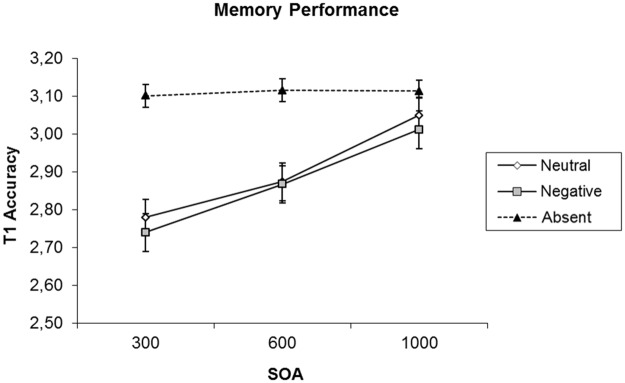
T1 accuracy by SOA in Experiment 2. Lines plot different picture conditions. The error bars show standard error of the mean.

After outlier exclusion, T1 recall accuracy was examined as a function of SOA and picture condition. This analysis revealed a significant effect of SOA on T1 recall, *F*(2, 46) = 13.19, *MSE* = 0.04, *p* < 0.001, *η*^*2*^ = 0.36, *BF*_*10*_ = 282.52. In addition, picture condition had a significant effect on T1 recall, *F*(2, 46) = 39.98, *MSE* = 0.03, *p* < 0.001, *η*^*2*^ = 0.64, *BF*_*10*_ = 45670000, and the interaction of SOA and picture condition was also significant, *F*(1, 23) = 2.85, *MSE* = 0.05, *p* = 0.03, *η*^*2*^ = 0.11 *BF*_*10*_ = 2.58. As can be seen in Fig 3, this interaction appeared to stem from the fact that T1 recall performance was stable across SOA in the picture-absent condition whereas it was impaired especially at short SOAs in the picture-present conditions, with no clear difference between conditions in which the picture was neutral or emotional. Consistent with this impression, the effect of SOA was non-significant in the picture-absent condition, *F*(2, 46) = 0.07, *MSE* = 0.02, *p* = 0.94, *BF*_*01*_ = 8.16, while an ANOVA contrasting the two picture-present conditions did reveal a main effect of SOA, *F*(2, 46) = 15.71, *MSE* = 0.05, *p* < 0.001, *η*^*2*^ = 0.41, *BF*_*10*_ = 22794.11. At the same time, there was no statistically significant difference between the neutral and emotional conditions, *F*(1, 23) = 0.08, *MSE* = 0.03, *p* = 0.78, *BF*_*01*_ = 5.59, and no interaction of SOA and the emotional contents of the picture, *F*(2, 46) = 0.22, *MSE* = 0.06, *p* = 0.81, *BF*_*01*_ = 6.85.

**Fig 4:**
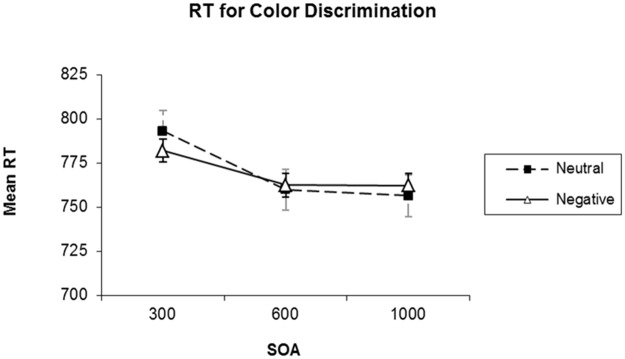
Mean response time for T2 (color discrimination) in picture present trials in of Experiment 2. Lines plot different picture conditions. The error bars show standard error of the mean.

In a second set of analyses, we examined performance for T2, using only the T2-present trials. For the analysis of effects of SOA and the emotional contents of the picture, the repeated measures ANOVA revealed no significant effects of SOA and picture type on accuracy, *F*(1.51, 34.82) = 0.26, *MSE* = 0.01, *p* = 0.71, *BF*_*01*_ = 11.49, and *F*(1, 23) = 0.25, *MSE* = 0.01, *p* = 0.62, *BF*_*01*_ = 4.84, respectively, and the interaction of these factors also failed to reach significance, *F*(1.79, 41.11) = 0.03, *MSE* = 0.01, *p* = 0.96, *BF*_*01*_ = 8.09. For the analysis of response times to T2, we only included trials in which the response was accurate. The main effect of SOA was significant, *F*(1.42, 32.59) = 3.70, *MSE* = 4580.44, *p* = 0.05, *η*^*2*^ = 0.14, *BF*_*10*_ = 4.63. In contrast, the main effect of picture type and the interaction of picture type and SOA interaction were not significant, *F*(1, 23) = 0.04, *MSE* = 845.80, *p* = 0.85, *BF*_*01*_ = 5.15 and *F*(1.99, 45.69) = 0.42, *MSE* = 2328.21, *p* = 0.66, *BF*_*01*_ = 6.21, respectively. To examine the effect of SOA in closer detail, we used pairwise comparisons of the different SOAs, using Bonferroni correction. These comparisons revealed a significant decrease in response time from the 300-ms SOA to the 600-ms SOA, *t(47)* = 2.98, *p* = 0.02, *BF*_*10*_ = 5.86, whereas the difference in response time between SOAs of 600 and 1000 ms, and between 300-ms SOA and the 1000-ms SOA were not significant, *t(47)* = 0.15, *p* = 1, *BF*_*01*_ = 6.28, and *t(47)* = 1.90, *p* = 0.20, *BF*_*01*_ = 0.66, respectively (see Fig 4).

### Comparison of Experiment 1 and Experiment 2

**Fig 5:**
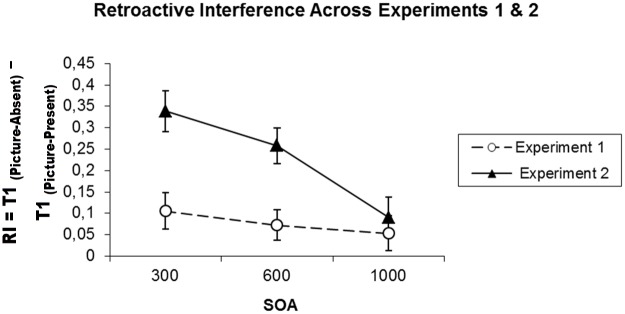
Mean retroactive interference observed in Experiment 1 and Experiment 2 as a function of SOA. The error bars show standard error of the mean.

In demonstrating a clear interaction effect of picture presence and SOA, the results from Experiment 2 differed from those of Experiment 1, wherein there was no task associated with the picture stimulus, and in which the analyses showed a detrimental effect of picture presence that was stable across SOA. To determine if the results were indeed significantly different in Experiments 1 and 2, we compared the interference effects produced by the presence of a picture as second stimulus in these experiments. The measure of interference was operationalized as the difference between the mean T1 accuracy for the picture-present condition and the mean T1 accuracy for the absent condition, for each experiment. To compare the results of Experiments 1 and 2, we used mixed-design ANOVA with experiment type coded as a between subjects factor, and SOA as a within-subject factor. The results revealed a significant effect of Experiment, *F*(1, 40) = 16.45, *MSE* = 0.04, *p* < 0.001, *η*^*2*^ = 0.29, *BF*_*10*_ = 27.55, and the interaction of Experiment by SOA closely approximated statistical significance, *F*(2, 80) = 3.05, *MSE* = 0.04, *p* = 0.053, *BF*_*01*_ = 0.57 with a significant effect for a linear contrast characterizing the fact that the difference in interference between experiments decreased across SOA, *F*(2, 80) = 6.69, *MSE* = 0.04, *p* = 0.002, *η*^*2*^ = 0.14, *BF*_*10*_ = 58.98 (see Fig 5).

## Discussion Experiment 2

In Experiment 2, we used the same design as in Experiment 1 with the implementation of a speeded 2-AFC task for the pictures that could appear following the to-be-remembered target. With this, we intended to replicate the retroactive interference effect observed by Nieuwenstein and Wyble [32], and we aimed to determine whether a negative emotional stimulus would produce stronger retroactive interference than a neutral stimulus when the stimulus is task-relevant. The analysis of T1 accuracy showed that retroactive interference was present for trials in which T2 was present. This was indicated by a reduction in T1 accuracy, which diminished as the onset of the T2 followed T1 at a later time. Importantly, however, there was no effect of the emotional content of T2 on T1 accuracy. Furthermore, accuracy and response times for the 2-AFC task did not depend on the emotional content of T2, indicating that the discrimination of the color of the outline frame was not affected by whether the picture it surrounded had a neutral or a negative emotional content.

While Experiment 2 replicated the results of Experiment 1 in showing no effect of the emotional contents of the picture trailing T1, the results did show a clear difference with the interference effects found in Experiment 1, where the presence of the same pictures led to a minor detrimental effect on T1 recall that was stable across SOA. In contrast, Experiment 2 showed a clear interaction effect, with the magnitude of the retro-active interference effect being much stronger and decreasing as a function of SOA. Taken together, these findings show that the interruption of T1 consolidation, which is evidenced by a decrease in retroactive interference with increasing SOA, is caused by the decision making and response selection requirements associated with a 2-AFC task and not by the mere appearance of a visual stimulus. In this regard, the results of the current study are consistent with the results of Nieuwenstein and Wyble [32] who found that the retroactive interference effect could not be explained in terms of masking as it only occurred when the second target was associated with a speeded 2-AFC task.

## General Discussion

In two experiments, we examined if a negative emotional picture can disrupt the consolidation of an earlier stimulus—a string of four letters that was shown for 80 ms and followed by a mask. The results showed that pictures with negative emotional content did not produce stronger interference than neutral pictures, and this lack of difference was seen regardless of whether the picture stimuli were task relevant. Examination of the associated Bayes factors confirmed that the null effects of emotion in Experiments 1 and 2 yielded moderate evidence in favor of the null hypothesis. In demonstrating a lack of effect of the emotional vs. neutral contents of the pictures used as second stimulus in both experiments, our results differ from earlier findings that did find attentional capture by emotional stimulus resulting in worse working memory performance (e.g., [25, 29, 31]). In the following sections, we discuss some possible (methodological) explanations for why we did not find effects of emotion in our study.

The present study was built on two principal assumptions. The first is that an emotional stimulus captures attention, for which ample evidence exists. The second assumption is that attentional capture contributes to retroactive interference. This second assumption may be challenged, particularly because the interference that was observed regardless of emotional content in Experiment 2 might also be attributed to task-switching instead. That is, in Experiment 2, in contrast to Experiment 1, a task switch had to be made for T2, thus possibly causing retro-active interference. Such switching costs have previously been predicted by Nieuwenstein and Wyble [32]. Even so, because attentional capture by an emotional second stimulus should still occur, one would expect its processing to speed up, and increase switching costs and thereby by cause more interference, and this was not observed. We must thus re-evaluate the other assumption of automatic capture by emotion; even if that has been repeatedly found in previous studies, it is possible that the present experiment differed in a critical way, such that capture was eliminated.

A first difference between our experiments and some of the earlier studies that found an effect of emotion (e.g., [25, 29, 31]), is the fact that we used a task in which the processing of the contents of the pictures may have been less likely to occur than in the attentional blink studies that did find effects of attentional capture by emotional distractors. In the latter set of studies, participants had to search for a rotated target picture that was embedded at an unpredictable position in a rapid sequence of distractor pictures. Therefore, each item within the stream could be the target, and therefore each picture had to be processed to determine whether it was a target or a distracter. In the current study, there was no need to process the contents of the picture following the to-be-remembered target and this difference in task demands may account for the different results we have found.

This account may also explain why associating the pictures with a 2-AFC task did not lead to stronger interference of emotional as compared to neutral pictures. Possibly, the reason why performing the 2-AFC task for the colored frame of the picture did not lead to stronger interference from emotional pictures was that the spatial location of the color frame was different than the picture, and perhaps participants could therefore ignore the contents of the picture while focusing on the outline frame and color. Indeed, it could be that the color discrimination task did not allow for an influence of the emotional contents of the pictures because of a difference in processing depth necessary for color discrimination and object identification. The results of Experiment 2 showed that the emotional vs. neutral content of the pictures did not influence accuracy or response times for the task associated with these pictures, thus lending further credence to the idea that the emotional pictures did not capture attention, as this would be expected to result in lower accuracy and longer response times for the color discrimination task.

Nevertheless, a hallmark of previous studies on attentional capture by emotional stimuli has been that it seems to occur even with stimuli whose contents are task-irrelevant, so that the processing of these stimuli should be minimal [25, 42]. On the basis of these previous findings, there appears to be little reason to assume that attentional capture should be entirely eliminated in the current design, only because the picture contents were not directly task relevant. There is also evidence, however, that emotional capture effects are modulated by task context [43–45], thus leaving the possibility that a similar (null) effect was elicited in the present study.

Finally, another reason for our failure to find any effects of emotion may lie in the selection of our participants. We advertised our study as a study with “potentially disturbing pictures”, so as to ensure that participants knew what they were signing up for. While this advertisement protected emotionally vulnerable participants from taking part, it could also have resulted in a selective sample of participants, who were less likely to react strongly to the emotional pictures. Indeed, it is of interest to note that it has been found that participants who score high on harm avoidance show stronger effects of negative emotional stimuli in an attentional blink paradigm [25].

### Conclusion

We failed to find any effects of emotional stimuli on reaction times and accuracy on a concurrent task, and on working memory consolidation of a preceding target. This lack of effect of emotional stimuli raises important questions for further research, including whether task-relevant emotional stimuli would cause effects in case the 2-AFC task we used would be modified in a way that the participants would be forced to process the contents of the pictures. It must also be noted that in real life, in conditions such as witnessing a crime involving a weapon, emotional stimuli are of much higher relevance for survival and may therefore exert stronger effects than an emotional picture on a PC screen, associated with a color discrimination task. In such real-life situations, the effects of an emotional encounter on ongoing working memory processes could be more drastic than our present results seem to suggest.

## Supporting Information

S1 Data Data of Experiment 1 and 2. (ZIP)

S1 Table Table of IAPS picture numbers used in Experiment 1 and 2. (DOCX)
