# Supplementary material for: Retro-Active Emotion: Do Negative Emotional Stimuli Disrupt Consolidation in Working Memory?
Source: PLoS One. 2017 Jan 19;12(1):e0169927. doi: 10.1371/journal.pone.0169927 (PMC5245818; doi:10.1371/journal.pone.0169927)
Supplement: S1 Table — (DOCX) [file pone.0169927.s002.docx]

***IAPS Pictures***

Neutral Pictures For Practice Trials [(4.39 < Valence < 5.42); (3.00 < Arousal < 3.07) N=14]

| 2102.JPG | 2850.JPG | 5500.JPG | 7034.JPG | 7055.JPG | 7052.JPG |
| --- | --- | --- | --- | --- | --- |
| 2221.JPG | 2870.JPG | 7009.JPG | 7038.JPG | 7056.JPG | 7160.JPG |
| 2381.JPG | 5120.JPG |  |  |  |  |

Negative Pictures For Practice Trials [(1.63 < Valence < 2.96); (6.01 < Arousal < 6.20) N=14]

| 3005.1.JPG | 6022.JPG | 6530.JPG | 9405.JPG | 9630.JPG | 9800.JPG |
| --- | --- | --- | --- | --- | --- |
| 4664.2.JPG | 6212.JPG | 9254.JPG | 9570.JPG | 9620.JPG | 9910.JPG |
| 6021.JPG | 6415.JPG |  |  |  |  |

Negative Experimental Pictures [(6.21 < Arousal < 7.35); (1.31 < Valence < 2.95); N=57]

| 2352.2.JPG | 3064.JPG | 3140.JPG | 6230.JPG | 6510.JPG | 9250.JPG |
| --- | --- | --- | --- | --- | --- |
| 2683.JPG | 3068.JPG | 3150.JPG | 6250.JPG | 6540.JPG | 9252.JPG |
| 2730.JPG | 3069.JPG | 3170.JPG | 6260.JPG | 6550.JPG | 9410.JPG |
| 2811.JPG | 3071.JPG | 3266.JPG | 6300.JPG | 6560.JPG | 9600.JPG |
| 3000.JPG | 3080.JPG | 3400.JPG | 6312.JPG | 6570.JPG | 9635.1.JPG |
| 3010.JPG | 3100.JPG | 3500.JPG | 6313.JPG | 6821.JPG | 9810.JPG |
| 3030.JPG | 3102.JPG | 3530.JPG | 6315.JPG | 6830.JPG | 9921.JPG |
| 3053.JPG | 3110.JPG | 3550.1.JPG | 6350.JPG | 6834.JPG |  |
| 3060.JPG | 3120.JPG | 6200.JPG | 6360.JPG | 8485.JPG |  |
| 3063.JPG | 3130.JPG | 6210.JPG | 6370.JPG | 9050.JPG |  |

Neutral Experimental Pictures [(1.72 < Arousal < 2.99); (4.03 < Valence < 5.93); N=57]

| 2038.JPG | 2880.JPG | 7004.JPG | 7050.JPG | 7161.JPG | 7235.JPG |
| --- | --- | --- | --- | --- | --- |
| 2190.JPG | 2890.JPG | 7006.JPG | 7053.JPG | 7175.JPG | 7490.JPG |
| 2393.JPG | 5130.JPG | 7010.JPG | 7059.JPG | 7179.JPG | 7491.JPG |
| 2397.JPG | 5390.JPG | 7020.JPG | 7060.JPG | 7185.JPG | 7700.JPG |
| 2440.JPG | 5510.JPG | 7025.JPG | 7080.JPG | 7187.JPG | 7705.JPG |
| 2480.JPG | 5520.JPG | 7030.JPG | 7090.JPG | 7205.JPG | 7950.JPG |
| 2570.JPG | 5530.JPG | 7031.JPG | 7100.JPG | 7217.JPG | 9360.JPG |
| 2580.JPG | 5731.JPG | 7035.JPG | 7110.JPG | 7224.JPG |  |
| 2620.JPG | 5740.JPG | 7040.JPG | 7140.JPG | 7233.JPG |  |
| 2840.JPG | 7000.JPG | 7041.JPG | 7150.JPG | 7234.JPG |  |
